# Density independent decline from an environmentally transmitted parasite

**DOI:** 10.1098/rsbl.2023.0169

**Published:** 2023-08-23

**Authors:** Scott Carver, Zachary M. Lewin, Leah G. Burgess, Vicky Wilkinson, Jason Whitehead, Michael M. Driessen

**Affiliations:** ^1^ Department of Biological Sciences, University of Tasmania, Tasmania, Australia; ^2^ Highland Conservation Pty Ltd, Australia; ^3^ Department of Natural Resources and Environment, Tasmania, Australia

**Keywords:** *Sarcoptes scabiei*, bare-nosed wombat, *Vombatus ursinus*, density dependence, environmental transmission, frequency dependent transmission

## Abstract

Invasive environmentally transmitted parasites have the potential to cause declines in host populations independent of host density, but this is rarely characterized in naturally occurring populations. We investigated (1) epidemiological features of a declining bare-nosed wombat (*Vombatus ursinus*) population in central Tasmania owing to a sarcoptic mange (agent *Sarcoptes scabiei*) outbreak, and (2) reviewed all longitudinal wombat–mange studies to improve our understanding of when host population declines may occur. Over a 7-year period, the wombat population declined 80% (95% CI 77–86%) and experienced a 55% range contraction. The average apparent prevalence of mange was high 27% (95% CI 21–34), increased slightly over our study period, and the population decline continued unabated, independent of declining host abundance. Combined with other longitudinal studies, our research indicated wombat populations may be at risk of decline when apparent prevalence exceeds 25%. This empirical study supports the capacity of environmentally transmitted parasites to cause density independent host population declines and suggests prevalence limits may be an indicator of impending decline-causing epizootics in bare-nosed wombats. This research is the first to test effects of density in mange epizootics where transmission is environmental and may provide a guide for when apparent prevalence indicates a local conservation threat.

## 1. Introduction

Invasive pathogens cause substantive impacts on wildlife populations and can have long-term consequences for population trajectories [1]. Theory often suggests host density plays an important role in disease outbreaks and population declines (density dependence), particularly for directly transmitted pathogens [2]. Hence, as a host population declines, pathogen prevalence may also gradually decline in accordance with diminished host contacts and the pathogen-specific time between infection and mortality. However, theory also suggests declines may occur independent of density where pathogen transmission is environmental [3–5]—that arising from free-living pathogen stages able to persist for lengths of time independent of the host and cause infection on host contact with fomites [6]. Hence, as host density declines, host encounter rates with fomites may remain unimpacted, meaning prevalence of disease remains high and a disease outbreak continues unabated [5]. In extreme cases, near or complete extirpation of host populations can result because of environmental transmission [7]. However, empirical examinations of density independence of environmentally transmitted pathogens are rare in naturally occurring populations.

Among the most invasive and impactful of mammalian parasites is the astigmatic mite *Sarcoptes scabiei*, which causes sarcoptic mange (termed scabies in humans) [1,8]. The parasite was dispersed associated with European colonialism and now forms a globally invasive panzootic (documented to infect at least 148 mammal species) [8]. Depending on host species *S. scabiei* has emerged or is emerging, with transmission spanning direct to environmental modes [9]. Where *S. scabiei* has emerged and become established, post-invasion epidemiological dynamics are variable, including endemic disease and outbreaks that cause local declines and extirpations [10,11]. Host density is often proposed as causally associated with *S. scabiei* induced population declines [10,12–15]. However, given variable transmission modes, declines may also occur independent of host density where transmission is dominated by environmental fomites. Density independent dynamics of environmentally transmitted *S. scabiei* are yet to be assessed for any host species.

Bare-nosed wombats (*Vombatus ursinus*) typify complex post-invasion dynamics of a virulent pathogen. Bare nosed wombats are large fossorial marsupial herbivores that are non-territorial, live largely solitary lives (meaning direct contacts are rare outside of mating), and share burrows asynchronously owing to burrow switching every 1–9 days [16,17]. *Sarcoptes scabiei* was introduced to Australia by Europeans and their domestic animals (likely multiple times since the late 1700s), with records of infection in wombats dating back over a century [18,19]. Sarcoptic mange is the most important disease affecting bare-nosed wombats, killing individuals it infects [20,21]. Exposure to *S. scabiei* occurs in burrows [22], with environmental transmission driven by burrow switching behaviours [17], which theory suggests it may or may not be linked with local density (based on above ground population counts [10]). Evidence suggests bare-nosed wombat populations predominantly sustain *S. scabiei* independent of other mammals [7], populations often persist in the presence of mange, and can also become disease free [10,23]. However, outbreaks driving gradual declines also occur [7], and epidemiological features associated with declines are rarely understood.

We describe a declining wombat population owing to sarcoptic mange and evaluate evidence of density independence and range contraction. We then compile and contrast all published literature on longitudinal wombat population studies where mange disease is reported, and examine if limits exist between mange and wombat population trajectories. Collectively, we characterize epidemiological features associated with variable post-invasion host–pathogen dynamics of an environmentally transmitted parasite.

## 2. Material and methods

### (a) Study site and surveys

This research was undertaken on private property in the central Tasmanian highlands (average elevation 650 m.a.s.l.). The site is characterized by highland *Poa* grassland, partially modified by stock grazing, surrounded by *Eucalyptus dalrympleana*, *E. pauciflora* and *E. delegatensis* dry forest and woodland [24]. Following detection of wombats showing signs of mange disease in 2014, surveys commenced in 2015 and were conducted through to 2022, with a gap in surveys between 2018 and 2020. Occurrence of mange disease at the site prior to 2014 is unreported, suggesting it was either absent or at low prevalence.

We undertook 29 survey trips, with most lasting 2–3 survey days to record counts of wombats and wombats with mange within our study area. Three surveys lasted 1 day and one lasted 4 days. On each occasion surveys of wombats and their signs of mange were undertaken by observation using established methods [7,25]. Briefly, surveys were conducted from late afternoon to dusk, walking a 4.4 km transect. Each observed wombat was assessed for clinical signs of mange (characteristic patterns of alopecia and hyperkeratosis [25]) using 10 × 42 magnification binoculars. For each observation, the location of the wombat and their mange status was recorded. In instances where the wombat fled or disappeared down a burrow before a mange assessment could be made, its location was recorded, but mange status was listed as unknown. Survey frequency varied across the 8-year study period, being most frequent in 2016 and 2020–2022, and can are broadly grouped into two defined time periods: 2015–2018 and 2020–2022.

In addition to dusk surveys, to ensure robust confirmation of population decline, we made efforts to survey wombats during the day (cooler temperatures at the site's elevation means wombats are often out during the day) and at a night by spotlight. As these additional surveys did not impact study findings and there was no perceivable overlap in counted individuals, all surveys on a given day were combined. We stress that wombat counts are necessarily an index of true population size, and counts have been shown to correlate among survey methods, suggesting they are representative [26].

We explicitly use the term ‘apparent prevalence’ to describe the relative proportion of the wombat population showing signs of sarcoptic mange. Diagnosis of *S. scabiei* infection is based on visual signs of disease (mainly alopecia and hyperkeratosis) that have been verified against other clinical diagnostic techniques [27]. Visual diagnosis of early stage *S. scabiei* infection is inherently variable, so estimates of mange prevalence are likely conservative in most instances [27].

Finally, we compiled all studies reporting longitudinal surveys of wombats and where mange disease was also reported, extracted study location, duration, number of wombats observed and apparent prevalence of mange. This evaluation of literature was undertaken to assess if there was any relationship between the apparent prevalence and wombat population trajectories.

### (b) Statistical analyses

All analyses were undertaken in R v. 4.0.3 using the ‘stats’, ‘rio’, ‘lubridate’, ‘stats4′, ‘arm’,’lme4, ‘adehabitatHR’, ‘rgda’ and ‘sp’ packages. Wombat data were used as wombats observed per survey day to account for minor variation in the number of surveys in a given day and because the same transect (4.4 km) was repeated. A generalized linear mixed model (GLMM) was used to evaluate changes in wombat abundance (our response variable) with time period (2015–2018 versus 2020–2022) and apparent prevalence of mange as predictor variables, and survey trip as a random effect. We excluded date from this analysis as it was confounded with time period, and the analytical outcomes were consistent. We additionally investigated whether the probability of a wombat being assigned as mange positive changed between the time periods using a GLMM with a binomial error distribution and survey trip as the random effect. For readers who prefer to see prevalence as the response variable, we also provide data and analyses in this representation (electronic supplementary material, S1)

Finally, we investigated if the distribution of wombats shifted between the two time periods by calculating and comparing the 100% minimum convex polygon (MCP) around wombat observation points in 2015–2018 versus 2020–2022. In preliminary analyses we considered a range of MCP sizes (100%, 90%, 80%, 70%) finding the same general conclusion was reached regardless (electronic supplementary material, S2).

## 3. Results

Across 29 survey trips, we undertook 60 individual surveys between 2015 and 2022. Twenty-two surveys were undertaken from 2015–2018 (12 trips), no surveys occurred from 2018–2020, and 38 surveys were undertaken from 2020–2022 (17 trips). A total of 182 wombats were observed (mean wombats per survey day 3.0, 95% CI 2.5–3.6). Sarcoptic mange was observed throughout the study with an average apparent prevalence of 26.9% (95% CI 20.8–33.7).

### (a) Characteristics of a declining wombat population

**Figure 1.**
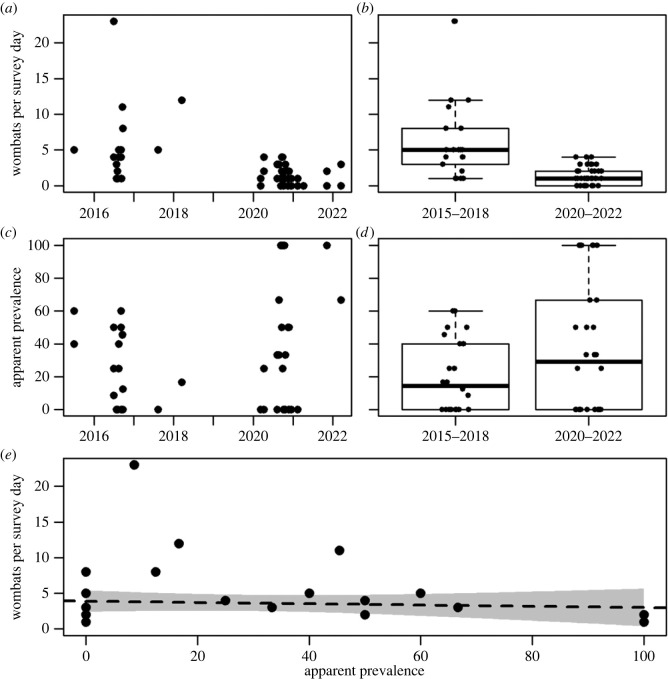
Declining wombats per survey day over time (*a*) and time period (*b*), increasing apparent prevalence of sarcoptic mange over time (*c*) and time period (*d*), and density independent relationship between wombats per survey day and the apparent prevalence of sarcoptic mange (*e*). Position of points jittered in (*b*) and (*d*) for visual clarity.

The average number of wombats observed per survey day declined by 80% (95% CI 77–86%) across the survey trips, from 5.9 (95% CI 5.3–6.4) during 2015–2018 to 1.4 (95% CI 0.8–2.0) during 2020–2022 (figure 1*a,b*). The apparent prevalence of mange for the two time periods increased from 21.7% (95% CI 15.2–29.3) to 39.6% (95% CI 27.2–53.1), respectively (figure 1*b,c*). It is notable that the smaller number of wombats per survey day during 2020–2022 meant estimates of apparent prevalence were inherently more variable (range 0–100% per individual survey, figure 1*c,d*).

The decline in the average number of wombats per survey was associated with time period (2015–2018 versus 2020–2022) and was unrelated to the apparent prevalence of mange (figure 1, Gaussian GLMM: time-period *F* = 9.83, *p* = 0.004; prevalence *F* = 2.41, *p* = 0.122). Notably, the probability of a wombat being assigned as sarcoptic mange positive did not decline as the number of wombats per survey reduced between the time periods, but rather increased (binomial GLMM: time-period *z* = 2.16, *p* = 0.031).

**Figure 2.**
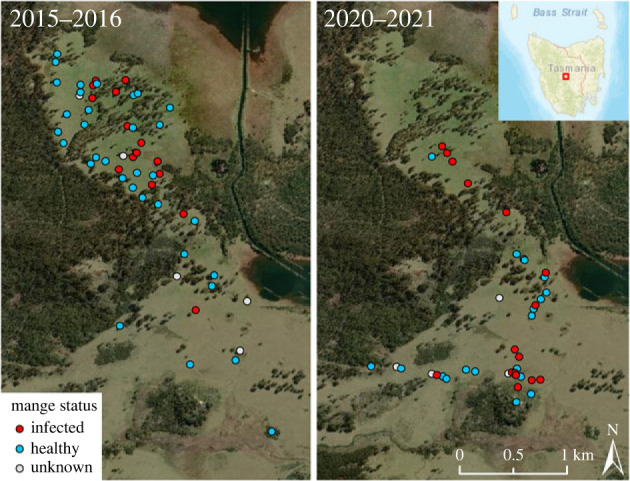
Reduction in the distribution of wombats from a site in central Tasmania between time periods 2015–2018 and 2020–2022, and mange disease status of wombats.

The distribution of wombats in the study area also contracted between the two study periods (figure 2). Proportional range contraction was estimated at 54.9%, based on a 100% MCP of 7.4 km^2^ in 2015–2018 to 4.5 km^2^ in 2020–2022. Range contraction was supported regardless of MCP size (electronic supplementary material, S2).

### (b) Longitudinal studies of wombat population trajectories in relation to mange

Including the present study, we identified nine published longitudinal studies documenting wombat population trajectories where mange disease was also documented: six from Tasmania and three from New South Wales (table 1). Study durations ranged from 2–9 years and the count of wombats from 79–1342. Of the nine studies, two documented mange outbreaks driving declining wombat populations, with average apparent prevalence estimates 27.7% and 33.9%. By contrast the average apparent prevalence of sarcoptic mange among remaining studies was 10.1% (range 0.0–24.9). Some inter-surveyor differences may exist in the diagnosis of mange across studies, particularly between the NSW and Tasmanian studies. Nevertheless, the general pattern among this limited study set was for population declines to be observed when mange prevalence was greater than 25% and stable when less than 25%. This interpretation may indicate the population at the NSW Wolgan Valley site to be at risk of decline (apparent prevalence 24.9%).

**Table 1.** Longitudinal studies of wombat populations documenting apparent prevalence of sarcoptic mange and population trajectories. TAS = Tasmania, NSW = New South Wales, NP = National Park.

location (state)	study timeframe	% mange prevalence (wombats surveyed)	population trajectory	reference
Bronte Park (TAS)	2015–2022	26.9 (182)	decline	present study
Narawntapu NP (TAS)	2010–2019	33.9 (103)	decline	Martin *et al*. [7]
Forestier Peninsula (TAS)	2017–2019	0.2 (419)	stable	Driessen *et al*. [22]
Cape Portland (TAS)	2017–2019	6.9 (1118)	stable	Driessen *et al*. [22]
Flinders Island (TAS)	2018–2019	5.0 (179)	stable	Driessen *et al*. [22]
Cradle Mountain (TAS)	2016–2019	0.0 (413)	stable	Driessen *et al*. [22]
Wolgan Valley (NSW)	2011–2017	24.9 (1342)	stable	Stannard *et al*. [28]
Badger Ground (NSW)	2015–2017	13.6 (176)	stable	Stannard *et al*. [28]
Eagle's Drift (NSW)	2015–2017	18.0 (137)	stable	Stannard *et al*. [28]

## 4. Discussion

Invasive environmentally transmitted parasites have potential to cause declines in host populations independent of host density, but the empirical features associated with variable host and pathogen dynamics are rarely studied in naturally occurring populations. Here, we investigated the epidemiological features of a declining wombat population owing to a sarcoptic mange outbreak, and contrasted longitudinal wombat–mange studies to help understand when host population declines are likely to occur. The study population declined by an average of 80% between 2015–2018 and 2020–2022, exhibiting a high (27%) and slightly increasing apparent prevalence of mange. The population decline was characterized by a 55% range reduction within the study area. Combined with other longitudinal studies, our research suggests wombat populations may be at risk of decline when the apparent prevalence exceeds 25%. This empirical study supports the capacity of environmentally transmitted parasites to cause density independent declines in host populations and suggests prevalence limits may be a useful indicator of impending epizootics.

This study represents the third formally documented decline of bare-nosed wombats owing to sarcoptic mange outbreaks. The first *S. scabiei*-driven decline for any host species was by Gray [29], who discussed the severe decline of bare-nosed wombats over a 5-year period in southern NSW. Although no empirical data were presented, descriptions suggest the decline was likely greater than 80%. More recently a detailed empirical description of *S. scabiei* causing a 94% decline of wombats in northern Tasmania was made by Martin *et al*. [7]. Like the present study, a host range reduction was also documented [7], and very low numbers of wombats continue to occur there [23]. Host population declines associated with *S. scabiei* are widespread in affected mammals, such as red-fox [30–32], kit fox [33], vicuña [34,35], ibex [36,37], chamois [38], grey wolf [11] and coyote [15]. Importantly, this research is the first to test for the effect of density in mange epizootics where transmission is principally environmental [9].

A general feature of *S. scabiei* outbreaks across host species is the extended timeframe over which epizootics take place. Mange outbreaks typically take years to spread through host populations [8], in contrast with many other important wildlife pathogens causing host declines (e.g. [39–41]), although not all (e.g. [42]). Whether a host population experiences a decline owing to *S. scabiei* may be governed by a range of factors, and indeed factors shaping transmission of *S. scabiei* vary across host species [9]. A recent study by Beeton *et al*. [10] suggested epidemiological outcomes of *S. scabiei* in bare-nosed wombat populations could be determined by host density, environmental survival of the parasite, host shedding of *S. scabiei* into burrows, and the rate at which wombats switch burrows. Our research suggests that once an outbreak is initiated, the abundance of bare-nosed wombats (measured as counts of individuals above ground) plays little role in modifying outbreak progression, pathogen prevalence or host decline. Similarly Martin *et al.* [7] also showed an outbreak in wombats continued unabated, regardless of host abundance. These findings are consistent with theory, suggesting environmentally transmitted pathogens can have density independent transmission and cause local population extirpations [2,4]. Further research on mechanisms underscoring environmental transmission of *S. scabiei* among bare-nosed wombats would be valuable. Absolute host abundance/density may not be the most critical factor, and a relative metric such as the ratio of burrows per wombat may be more useful, as this could dictate the extent of shared space use and probability of environmental exposure [17]. Given wombats have relatively small and fixed home ranges, the combination of burrow switching, ratio of burrows per wombat, and home range stability may explain why declines continue when host densities are low, and reciprocally, why the apparent prevalence of manage can remain stable in some populations over time [23,43].

A potentially valuable outcome of this study is the relationship between apparent prevalence of mange and whether a wombat population remains stable or declines. While the apparent prevalence threshold of 25% we identified is necessarily tentative because of the relatively small number of studies available, it does provide a reference from which other studies may conservatively consider a warning of local conservation issues. It is also important to acknowledge apparent prevalence is a conservative estimate of true *S. scabiei* prevalence in wombat populations [27]. Under-diagnosis rates are poorly understood, particularly at early stages of infection, and some research suggests ‘true’ prevalence estimates could be 25% higher than apparent prevalence during epizootics [27]. Similarly, population level prevalence estimates may vary among observers, owing to expertise in diagnosing clinical signs. This contributes to some caution in our interpretation of mange prevalence and host decline relationships. However, among the Tasmanian studies a similar consistency of researcher training has been used, giving some confidence that the 25% value may be reasonable.

## Data Availability

Data supporting findings of this study are available from the Dryad Digital Repository: https://doi.org/10.5061/dryad.pk0p2ngsz [44]. The data are provided in the electronic supplementary material [45].

## References

[1] Tompkins DM, Carver S, Jones ME, Krkošek M, Skerratt LF. 2015 Emerging infectious diseases of wildlife: a critical perspective. Trends Parasitol. **31**, 149-159. (10.1016/j.pt.2015.01.007) 25709109

[2] Keeling MJ, Rohani P. 2008 Modeling infectious diseases in humans and animals. Princeton, NJ: Princeton University Press.

[3] Hoyt JR et al. 2023 Reducing environmentally mediated transmission to moderate impacts of an emerging wildlife disease. J. Appl. Ecol. **60**, 923-933. (10.1111/1365-2664.14371)

[4] Ryder JJ, Miller MR, White A, Knell RJ, Boots M. 2007 Host–parasite population dynamics under combined frequency- and density-dependent transmission. Oikos **116**, 2017-2026. (10.1111/j.2007.0030-1299.15863.x)

[5] Hoyt JR et al. 2020 Environmental reservoir dynamics predict global infection patterns and population impacts for the fungal disease white-nose syndrome. Proc. Natl Acad. Sci. USA **117**, 7255-7262. (10.1073/pnas.1914794117) 32179668 PMC7132137

[6] Park AW. 2012 Infectious disease in animal metapopulations: the importance of environmental transmission. Ecol. Evol. **2**, 1398-1407. (10.1002/ece3.257) 22957148 PMC3434925

[7] Martin AM, Burridge CP, Ingram J, Fraser TA, Carver S. 2018 Invasive pathogen drives host population collapse: effects of a travelling wave of sarcoptic mange on bare-nosed wombats. J. Appl. Ecol. **55**, 331-341. (10.1111/1365-2664.12968)

[8] Escobar LE et al. 2022 Sarcoptic mange: an emerging panzootic in wildlife. Transbound. Emerg. Dis. **69**, 927-942. (10.1111/tbed.14082) 33756055

[9] Browne E, Driessen MM, Cross PC, Escobar LE, Foley J, López-Olvera JR, Niedringhaus KD, Rossi L, Carver S. 2022 Sustaining transmission in different host species: the emblematic case of *Sarcoptes scabiei*. BioScience **72**, 166-176. (10.1093/biosci/biab106)

[10] Beeton NJ, Carver S, Forbes LK. 2019 A model for the treatment of environmentally transmitted sarcoptic mange in bare-nosed wombats (*Vombatus ursinus*). J. Theor. Biol. **462**, 466-474. (10.1016/j.jtbi.2018.11.033) 30502410

[11] Niedringhaus KD, Brown JD, Sweeley KM, Yabsley MJ. 2019 A review of sarcoptic mange in North American wildlife. Int. J. Parasitol.: Parasites Wildl. **9**, 285-297. (10.1016/j.ijppaw.2019.06.003) 31304085 PMC6599944

[12] Almberg ES, Cross PC, Dobson AP, Smith DW, Hudson PJ. 2012 Parasite invasion following host reintroduction: a case study of Yellowstone's wolves. Phil. Trans. R. Soc. B **367**, 2840-2851. (10.1098/rstb.2011.0369) 22966139 PMC3427562

[13] Iacopelli F, Fanelli A, Tizzani P, Berriatua E, Prieto P, Martínez-Carrasco C, León L, Rossi L, Candela MG. 2020 Spatio-temporal patterns of sarcoptic mange in red deer and Iberian ibex in a multi-host natural park. Res. Vet. Sci. **128**, 224-229. (10.1016/j.rvsc.2019.11.014) 31837510

[14] Kido N, Itabashi M, Takahashi M, Futami M. 2013 Epidemiology of sarcoptic mange in free-ranging raccoon dogs (*Nyctereutes procyonoides*) in Yokohama, Japan. Vet. Parasitol. **191**, 102-107. (10.1016/j.vetpar.2012.07.026) 22902260

[15] Pence DB, Windberg LA. 1994 Impact of a sarcoptic mange epizootic on a coyote population. J. Wildl. Manage. **58**, 624-633. (10.2307/3809675)

[16] Triggs B. 2009 Wombats. Collingwood, Australia: CSIRO Publishing.

[17] Martin AM, Richards SA, Fraser TA, Polkinghorne A, Burridge CP, Carver S. 2019 Population-scale treatment informs solutions for control of environmentally transmitted wildlife disease. J. Appl. Ecol. **56**, 2363-2375. (10.1111/1365-2664.13467)

[18] Fraser TA, Charleston M, Martin A, Polkinghorne A, Carver S. 2016 The emergence of sarcoptic mange in Australian wildlife: an unresolved debate. Parasites Vectors **9**, 316. (10.1186/s13071-016-1578-2) 27255333 PMC4890250

[19] Fraser TA, Holme R, Martin A, Whiteley P, Montarello M, Raw C, Carver S, Polkinghorne A. 2019 Expanded molecular typing of *Sarcoptes scabiei* provides further evidence of disease spillover events in the epidemiology of srcoptic mange in Australian marsupials. J. Wildl Dis. **55**, 231-237. (10.7589/2018-04-101) 30096035

[20] Martin A, Skerratt L, Carver S. 2017 Sarcoptic mange in Australian wildlife. Fact sheet for Wildlife Health Australia. See https://wildlifehealthaustralia.com.au/FactSheets.aspx.

[21] Old JM, Sengupta C, Narayan E, Wolfenden J. 2018 Sarcoptic mange in wombats—a review and future research directions. Transboundary Emerg. Dis. **65**, 399-407. (10.1111/tbed.12770) 29150905

[22] Browne E, Driessen MM, Ross R, Roach M, Carver S. 2021 Environmental suitability of bare-nosed wombat burrows for *Sarcoptes scabiei*. Int. J. Parasitol.: Parasites Wildl. **16**, 37-47. (10.1016/j.ijppaw.2021.08.003) 34434693 PMC8374697

[23] Driessen MM, Dewar E, Carver S, Gales R. 2022 Conservation status of common wombats in Tasmania I: incidence of mange and its significance. Pacific Conserv. Biol. **28**, 103-114. (10.1071/PC21007)

[24] Department of Natural Resources and Environment Tasmania, Tasmanian Government. 2020. Tasmanian Vegetation Monitoring and Mapping Program (including TASVEG). See https://nre.tas.gov.au/conservation/development-planning-conservation-assessment/planning-tools/monitoring-and-mapping-tasmanias-vegetation-(tasveg) (accessed 28 January 2023).

[25] Simpson K, Johnson CN, Carver S. 2016 *Sarcoptes scabiei*: the mange mite with mighty effects on the common wombat (*Vombatus ursinus*). PLoS One **11**, e0149749. (10.1371/journal.pone.0149749) 26943790 PMC4778766

[26] Carver S, Charleston M, Hocking G, Gales R, Driessen MM. 2021 Long-term spatiotemporal dynamics and factors associated with trends in bare-nosed wombats. J. Wildl. Manage. **85**, 449-461. (10.1002/jwmg.22014)

[27] Fraser TA, Martin A, Polkinghorne A, Carver S. 2018 Comparative diagnostics reveals PCR assays on skin scrapings is the most reliable method to detect *Sarcoptes scabiei* infestations. Vet. Parasitol. **251**, 119-124. (10.1016/j.vetpar.2018.01.007) 29426467

[28] Stannard HJ, Wolfenden J, Hermsen EM, Vallin BT, Hunter NE, Old JM. 2020 Incidence of sarcoptic mange in bare-nosed wombats (*Vombatus ursinus*). *Aust*. *Mammal*. **2**, 85–95. (10.1071/AM20001)

[29] Gray DF. 1937 Sarcoptic mange affecting wild fauna in New South Wales. Aust. Vet. J. **13**, 154-155. (10.1111/j.1751-0813.1937.tb04110.x)

[30] Willebrand T, Samelius G, Walton Z, Odden M, Englund J. 2022 Declining survival rates of red foxes *Vulpes vulpes* during the first outbreak of sarcoptic mange in Sweden. Wildl. Biol. **2022**, e01014. (10.1002/wlb3.01014)

[31] Bornstein S, Morner T, Samuel WM. 2001 *Sarcoptes scabiei* and sarcoptic mange. In Parasitic diseases of wild mammals (eds WM Samuel, MJ Pybus, AA Kocan), 2nd edn. London, UK: Manson Publishing.

[32] Uraguchi K, Ueno M, Iijima H, Saitoh T. 2014 Demographic analyses of a fox population suffering from sarcoptic mange. J. Wildl. Manage. **78**, 1356-1371. (10.1002/jwmg.794)

[33] Cypher BL, Rudd JL, Westall TL, Woods LW, Stephenson N, Foley JE, Richardson D, Clifford DL. 2017 Sarcoptic mange in endangered kit foxes (*Vulpes macrotis mutica*): case histories, diagnoses, and implications for conservation. J. Wildl Dis. **53**, 46-53. (10.7589/2016-05-098) 27669012

[34] Ferreyra HDV, Rudd J, Foley J, Vanstreels RET, Martín AM, Donadio E, Uhart MM. 2022 Sarcoptic mange outbreak decimates South American wild camelid populations in San Guillermo National Park, Argentina. PLoS One **17**, e0256616. (10.1371/journal.pone.0256616) 35061672 PMC8782313

[35] Monk JD et al. 2022 Cascading effects of a disease outbreak in a remote protected area. Ecol. Lett. **25**, 1152-1163. (10.1111/ele.13983) 35175672

[36] León-Vizcaíno L, Ruíz de Ybáñez MR, Cubero MJ, Ortíz JM, Espinosa J, Pérez L, Simón MA, Alonso F. 1999 Sarcoptic mange in Spanish ibex from Spain. J. Wildl Dis. **35**, 647-659. (10.7589/0090-3558-35.4.647) 10574523

[37] Perez JM, Ruiz-Moreno I, Granados JE, Soriguer RC, Fandos P. 1997 The dynamics of sarcoptic mange in the ibex population of Sierra Nevada in Spain—influence of climatic factors. J. Wildl. Res. **2**, 86-89.

[38] Buzan EV, Bryja J, Zemanová B, Kryštufek B. 2013 Population genetics of chamois in the contact zone between the Alps and the Dinaric Mountains: uncovering the role of habitat fragmentation and past management. Conserv. Genet. **14**, 401-412. (10.1007/s10592-013-0469-8)

[39] Langwig KE, Frick WF, Hoyt JR, Parise KL, Drees KP, Kunz TH, Foster JT, Kilpatrick AM. 2016 Drivers of variation in species impacts for a multi-host fungal disease of bats. Phil. Trans. R. Soc. B **371**, 20150456. (10.1098/rstb.2015.0456) 28080982 PMC5095535

[40] Kilpatrick AM, Briggs CJ, Daszak P. 2010 The ecology and impact of chytridiomycosis: an emerging disease of amphibians. Trends Ecol. Evol. **25**, 109-118. (10.1016/j.tree.2009.07.011) 19836101

[41] LaDeau SL, Kilpatrick AM, Marra PP. 2007 West Nile virus emergence and large-scale declines of North American bird populations. Nature **447**, 710-713. (10.1038/nature05829) 17507930

[42] Bell BD, Carver S, Mitchell NJ, Pledger S. 2004 The dramatic decline of a New Zealand endemic: how and why did populations of Archey's frog *Leiopelma archeyi* crash over 1996–2001? Biol. Conserv. **120**, 193-203.

[43] Burgess LG, Richards SA, Driessen MM, Wilkinson V, Amin RJ, Carver S. 2023 Fine-scale landscape epidemiology: sarcoptic mange in bare-nosed wombats (*Vombatus ursinus*). Transbound. Emerg. Dis. **2023**, 2955321. (10.1155/2023/2955321) PMC12016856 40303715

[44] Carver S, Lewin ZM, Burgess LG, Wilkinson V, Whitehead J, Driessen MM. 2023 Data from: Density independent decline from an environmentally transmitted parasite. Dryad Digital Repository. (10.5061/dryad.pk0p2ngsz) PMC10444343 37607579

[45] Carver S, Lewin ZM, Burgess LG, Wilkinson V, Whitehead J, Driessen MM. 2023 Density independent decline from an environmentally transmitted parasite. Figshare. (10.6084/m9.figshare.c.6793675) PMC10444343 37607579

